# Age dependent contribution of entry via the CSF to the overall brain entry of small and large hydrophilic markers

**DOI:** 10.1186/s12987-022-00387-z

**Published:** 2022-11-14

**Authors:** Fiona Qiu, Yifan Huang, Norman R. Saunders, Mark D. Habgood, Katarzyna M. Dziegielewska

**Affiliations:** Department of Neuroscience, Central Clinical School, Monash University, Melbourne, VIC Australia

**Keywords:** Cerebrospinal fluid, CSF, Brain, Development, Sucrose, Dextran, Blood brain barrier, BBB, Blood CSF barrier, BCSFB, Residual vascular space, RVS

## Abstract

**Background:**

Apparent permeability of the blood brain barrier to hydrophilic markers has been shown to be higher in the developing brain. Apart from synthesis in situ*,* any substance detected in the brain parenchyma can originate from two sources: directly through blood vessels of brain vasculature and/or indirectly by entry from the cerebrospinal fluid (CSF) after transfer across the choroid plexuses. The relative quantitative contribution of these two routes to the overall brain entry remains unclear.

**Methods:**

In rats at embryonic day 16, 19 and postnatal day 4 and young adults, a small (sucrose, mw. 342 Da) or a large (dextran, mw. 70 kDa) radiolabelled hydrophilic marker was injected intravenously for very short periods of time (30 s to 5 min) before collection of plasma, cerebrospinal fluid (CSF) and brain samples. Results are presented as concentration ratios between radioactivity measured in CSF or brain and that in plasma (%).

**Results:**

The dextran brain/plasma ratio five minutes post injection was similar (2–4%) from E16 to adulthood whereas the sucrose brain/plasma ratio was significantly higher in fetal brains, but was comparable to dextran values in the adult. Sucrose CSF/plasma ratios were also significantly higher in fetal animals and decreased with age. In very short experiments involving fetal animals, entry of sucrose into the CSF after only 30 s was similar to that of dextran and both markers showed similar brain/plasma ratios.

**Conclusions:**

In the developing brain the apparent higher brain entry of a small hydrophilic marker such as sucrose can be attributed to its higher entry into the CSF and subsequent diffusion into the brain. By contrast, movement of a larger marker like 70 kDa dextran is restricted firstly by choroid plexus epithelial tight junctions and secondly by specialised junctions in the neuroependymal interface between the CSF and brain. Brain/plasma ratios of 70 kDa dextran were similar in fetal and adult rats. Therefore 70 kDa dextran should be considered an appropriate marker if brain residual vascular space is to be measured, especially in younger animals.

**Supplementary Information:**

The online version contains supplementary material available at 10.1186/s12987-022-00387-z.

## Background

Entry of molecules from blood into the central nervous system (CNS) is limited by selectively permeable barriers that include the blood–brain barrier proper (BBB) and the blood-cerebrospinal fluid barrier, BCSFB [1]. Throughout development, tight junctions between endothelial cells of brain capillaries (BBB) and epithelial cells of the choroid plexus (BCSFB) have been shown to confer effective intercellular restriction to the passive diffusion of large (proteins) and small hydrophilic molecules in spite of some changes in the molecular structure of the junctions [2–4]. At the BCSFB, entry of these substances into CSF has been proposed to take place transcellularly via a small subpopulation of specialised choroid plexus epithelial cells [5–8]. Once in CSF, these molecules are able to diffuse into the brain itself across the brain-CSF interface [6]. However in the developing brain, the CSF and brain compartments are separated by specialised single-strand junctions, called strap junctions, between cells forming the neuroepithelium. These junctions are morphologically distinct from tight junctions; they can only be defined clearly by freeze fracture. With this technique their appearance is unique amongst different types of intercellular junction [9]. These specialised junctions restrict passive diffusion based on molecular size and progressively disappear with brain development to be replaced by the non-restrictive gap junctions in the more mature ependyma, which allow unrestricted permeation regardless of molecular size [10, 11].

Experimental approaches to determine the extent of transfer of molecules, including tracers and therapeutics, across brain barriers have mostly involved administering test substances into the circulation and measuring their levels in the brain and CSF after a period of time. Results are usually expressed as ratios between concentrations estimated in blood plasma and those in brain tissues [12]. However, any substance measured in the brain parenchyma can in fact originate from two sources: direct entry into the brain via the cerebral blood vessels or indirectly via the CSF after transfer across the choroid plexuses [10]. There has been a longstanding discussion of the relative quantitative contribution of these two routes of brain entry [13] but distinguishing them proved to be difficult in in vivo experiments. Such information is important, especially in the developing brain. In the adult brain, using the technique of ventriculo-cisternal perfusion it has been shown that a variety of molecules enter the brain from the CSF to a different extent, with decrease in concentrations of the tracer from caudate nucleus outward. [14–16]. Because of the turnover of CSF in the adult brain it has been generally found that much of test markers added to the perfusion fluid is lost in the drainage of the CSF and only a small but variable amount enters the brain. A comparison of exposure of the brain to ^14^C-sucrose applied intravenously, via the CSF or a combination of the two showed that the combination resulted in the highest level of this marker in the brain [17]. Such observations accord with results from studies using ^131^I [16, 18]. These findings indicate that entry into the brain can occur both via the cerebral blood vessels and via choroid plexuses and CSF, but does not resolve their relative contributions. The situation in the developing brain is different. The turnover of CSF is very low [6] and there is a restriction on passage from CSF to brain for hydrophilic molecules because of the presence of specialised junctional complexes termed strap junctions between the neuroependymal cells (neuroepithelium) lining the ventricles [9].

For any brain permeability study, levels of test compound in brain tissue samples also include a quantity of test compound that remains inside the lumen of blood vessels within the sample and this blood contamination thus increases the estimate of entry into the brain. Here we use the term residual vascular space (RVS) to refer to the total intravascular volume of blood that remains trapped in blood vessels. Morphological studies based on counting the number of capillaries per mm^2^ of brain or measuring their length in fixed brains concluded that vascularisation of cortex in fetal and early postnatal rats is relatively limited, but shows dramatic increases during the second week of life and approaching adult levels by about postnatal day P20 [19–24]. Only a few studies have determined RVS in vivo using tracers during development and the limited data available suggest that this may be about 1–3% in early and late fetal sheep brain [25], which is very similar to that reported for adult brains [26, 27].

In this study we have extended the range of brain development from fetal to adult rats. We describe the entry of a small (sucrose, mw. 342 Da) and a large (dextran, mw. 70 kDa) hydrophilic marker into the brain and CSF of rats from embryonic day E16 to adulthood following intravenous injections of either marker for a very short period of time (30 s to 5 min). Comparison between the two markers over this time allows for demarcation of permeability properties of the two routes of entry based on passive diffusion since a molecule the size of 70 kDa dextran would not reach the CSF to any significant degree even in 5 min, whereas a faster diffusing small molecule like sucrose is likely to [28]. In turn, the difference in their apparent entries into the brain could then be correlated with their presence in the CSF. Thus the apparent increased entry of sucrose into the fetal brain can be explained by the contribution of brain entry from the CSF.

## Methods

### Animals & ethics

Sprague–Dawley rats used in this study were supplied by the Biological Research Facility at The University of Melbourne, Australia. Animals were kept in a 12-h light/dark cycle and were provided with free access to food and water. Experiments were conducted in embryonic (E) day 16 and 19 fetuses from time-mated females, as well as postnatal (P) day 4 and 7 week-old adult rats in accordance with the National Health and Medical Research Committee guidelines. All procedures were approved by The University of Melbourne Ethics Committee (Ethics ID: 1714344.1). At all age groups animals came from at least two separate litters. All experiments were conducted in the morning.

### Surgery & sample preparation

Prior to surgical procedures, all animals were deeply anesthetised with intraperitoneally injected urethane (1.0 g/kg for P4 pups, 2.0 g/kg for adults and pregnant dams). In postnatal rats (P4 and adults), the left femoral vein was exposed. A trace amount of ^3^H-dextran (American Radiolabelled Chemicals Inc.) or ^14^C-sucrose (PerkinElmer Inc.), in sterile physiological saline (0.9% NaCl), was injected intravenously using glass micropipettes or insulin syringes. In pregnant animals, a tracheal catheter was inserted to maintain a clear airway and a small abdominal incision was made to exteriorise the uterine horn. Injections were administered directly to individual fetuses via the umbilical vein using glass micropipettes. Volume of injections were kept to a minimum (< 5% of the total estimated blood volume). After 30 s, 1 or 5 min, animals were exsanguinated by cardiac puncture into the right ventricle. CSF samples were collected from the *cisterna magna* except in E16 pups where it was sampled from the lateral ventricles due to size constrains. Cortex and brainstem samples were collected. Due to the very small sizes of fetal rats, CSF and brain samples were collected from separate fetuses, but within the same litter. In all postnatal rats CSF, brain and blood samples were obtained from the same animal.

Sample preparation for radioactivity measurement has been previously described [29–31]. Briefly, plasma was separated by centrifuging blood samples at 1957xg for 5 min. Weighed brain samples (< 40 mg) were solubilised in 0.5 ml of Soluene (PerkinElmer Inc.) overnight and neutralised by acetic acid. Five ml of scintillation fluid (PerkinElmer Inc.) was added to all samples before radioactivity (Disintegrations per minute, DPM) was counted on a liquid scintillation counter (Tri-Carb 4910 TR, PerkinElmer Inc.). Background radioactivity counts (DPM) were established using blank tissue samples from control rats and these were subtracted from the experimental sample counts. Results were normalised as radioactivity per μl or mg of sample and expressed as a concentration ratio in relation to the corresponding plasma radioactivity as follows:

$$CSF or \,Brain \,to \,plasma \,ratio (\mathrm{\%})=\frac{CSF DPM/\mu l \,or\, brain\, DPM/mg}{Plasma \,DPM/\mu l} \times 100\mathrm{\%}$$

Tables 1 & 2 show the number of animals used and samples of brain and CSF obtained for the dextran and sucrose experiments.

**Table 1:** Number of animals used for dextran experiments

	Cortex (n)	Brainstem (n)	CSF (n)	Total animal number
E16–30 s	3	–	2	5
E16–5 min	4	4	3	7
E19–30 s	3	–	4	7
E19–1 min	–	–	4	4
E19–5 min	6	6	4	10
P4–5 min	4	4	4	4
Adult–5 min	4	4	4	4

Number of animals used for each experimental group. For fetal experiments, brain regions and CSF were collected from separate individuals due to small size, in all postnatal rats brain and CSF samples were collected from the same animal; (n) indicates numbers of samples

**Table 2:** Number of animals used for sucrose experiments

	Cortex (n)	Brainstem (n)	CSF (n)	Total animal number
E16–30 s	3	–	3	6
E16–5 min	3	3	3	6
E19–30 s	3	–	4	7
E19–1 min	–	–	4	4
E19–5 min	5	5	3	8
P4–5 min	6	6	6	6
Adult–5 min	3	3	3	3

Number of animals used for each experimental group. For fetal experiments, brain regions and CSF were collected from separate individuals due to small size, in all postnatal rats brain and CSF samples were collected from the same animal; (n) indicates numbers of samples

### Randomisation & statistics

Animals were assigned randomly from the Animal Facility to receive either one of the two markers. Both female and male pups were used at E16, E19 and P4. Data analyses were performed using Prism (GraphPad Software Inc.); statistical differences between the two markers were determined using unpaired Student t tests with F tests and one-way ANOVA (analysis of variance) with Tukey’s multiple comparisons test to compare changes across all 4 age groups. A p-value of 0.05 or less was considered statistically significant. Results are presented as mean ± standard deviation (SD).

## Results

Results are presented as ratios between radioactivity in CSF or brain and radioactivity in plasma and expressed as CSF/plasma or brain/plasma concentration ratios (%) in samples collected 5 min following intravenous injection of radiolabelled tracers. All numerical values are reported in Additional file 1: Tables S1 & S2.

### Dextran

**Fig. 1:**
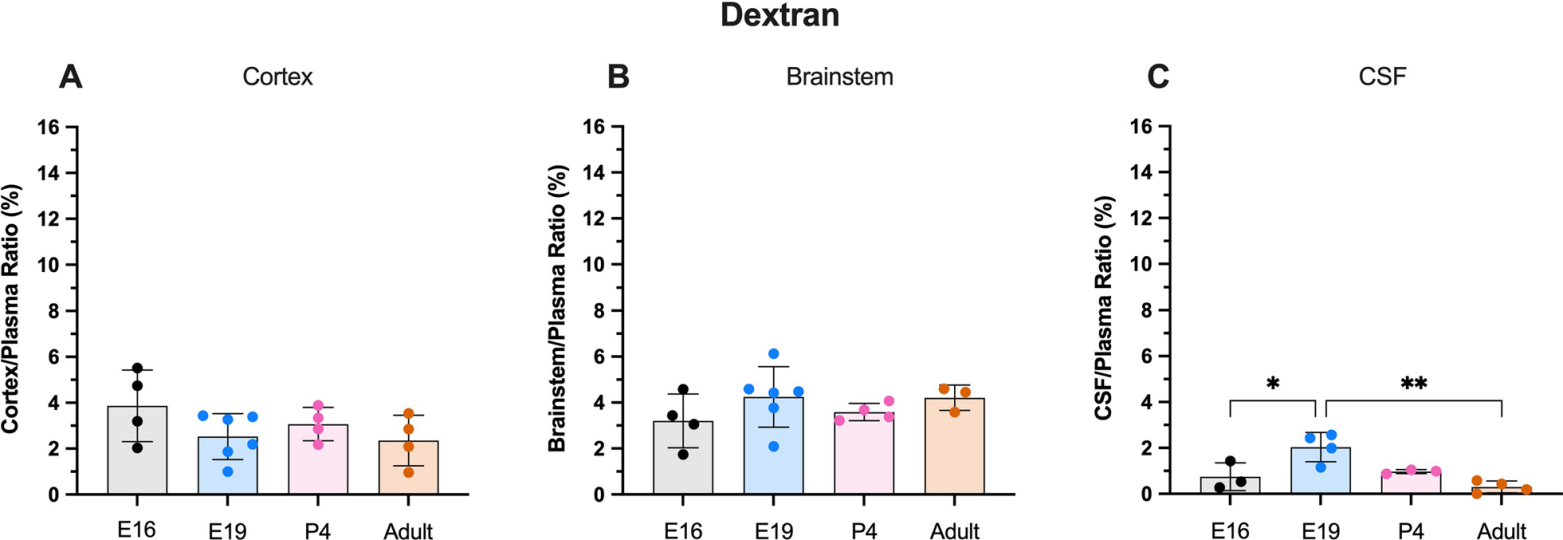
Dextran brain and CSF concentration ratios. **A** Cortex/plasma, **B** Brainstem/plasma and **C** CSF/plasma concentration ratios (%) in E16, E19, P4 and adult rats. Samples collected 5 min following i.v. injection of 70 kDa ^3^H-dextran. Note no difference in brain ratios between the ages (**A** & **B**). Each point represents result from a single animal. Mean ± SD; n = 3–6. *p < 0.05, **p < 0.01

Five minutes after i.v. injection of ^3^H-70 kDa dextran, brain/plasma concentration ratios in both brain regions (cortex and brainstem) ranged between 2–4% with no significant difference between any age groups (Fig. 1A & B). The cortex/plasma concentration ratios were 3.8 ± 1.6% in E16 animals, 2.5 ± 1.0% in E19, 3.1 ± 0.6% in P4 and 2.4 ± 1.0% in adults. The brainstem ratios were 3.2 ± 1.2% (E16), 4.3 ± 1.3% (E19), 3.6 ± 0.3% (P4) and 4.2 ± 0.5% (adult). Entry into the CSF was lower than into the brain at all ages and remained below 1% except for E19 where the ratios approached 2.0 ± 0.6% (Fig. 1C).

### Sucrose

**Fig. 2:**
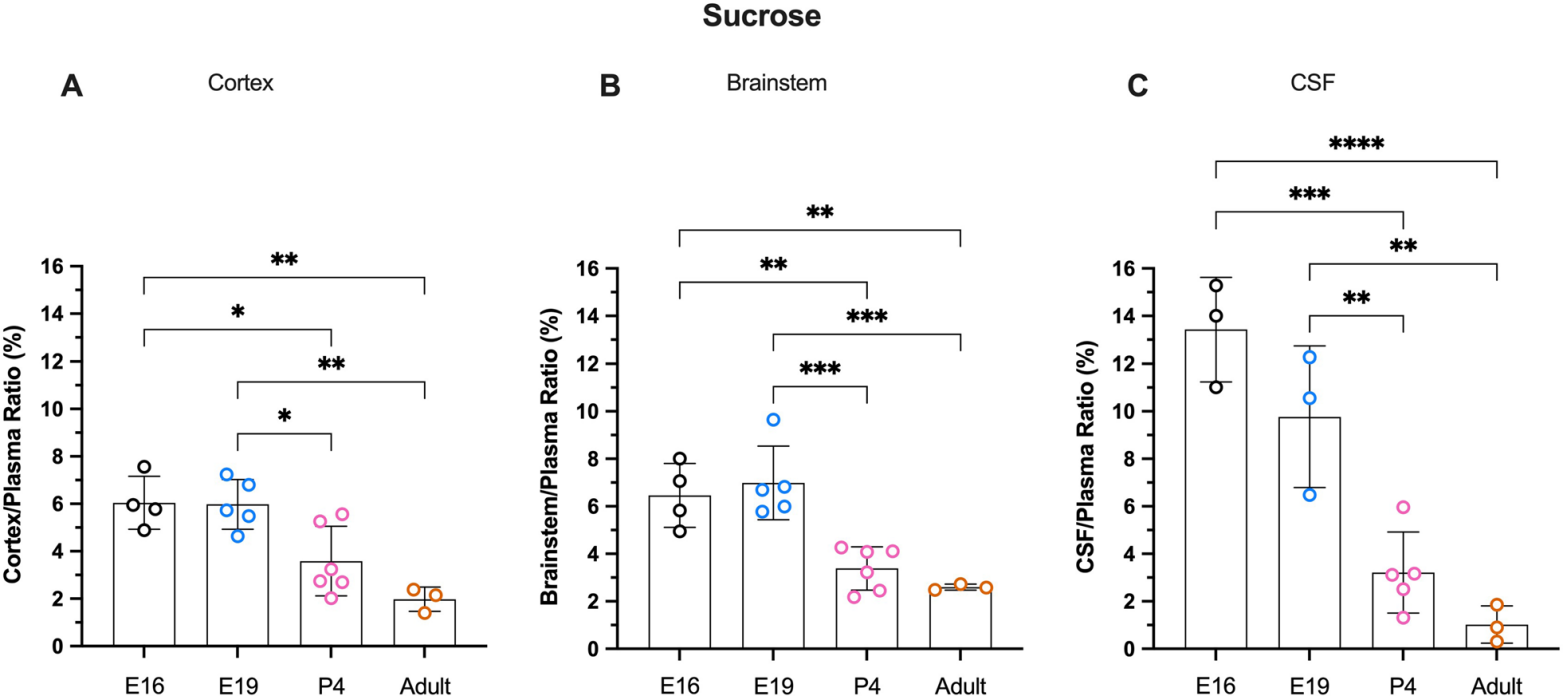
Sucrose brain and CSF concentration ratios. **A** Cortex/plasma, **B** Brainstem/plasma and **C** CSF/plasma concentration ratios (%) in E16, E19, P4 and adult rats. Samples collected 5 min following i.v. injection of ^14^C-sucrose. Each point represents result from a single animal. Mean ± SD; n = 3–6. *p < 0.05, **p < 0.01, ***p < 0.001, ****p < 0.0001

In contrast to dextran, sucrose concentration ratios appeared to decrease with age in both brain regions and in the CSF. The cortex/plasma ratio of sucrose was highest in fetal brains (6.0 ± 1.1% and 6.0 ± 0.9%; E16 and E19 respectively) and decreased significantly in P4 animals to 3.6 ± 1.3% (p < 0.05). There was a further small decrease in adults (2.0 ± 0.4%) but the difference compared to P4 was not statistically significant (p = 0.12**, **Fig. 2A). A similar age-related trend was observed in the brainstem (Fig. 2B) with ratios in fetal animals (6.5 ± 1.3% and 7.0 ± 1.4%; E16 and E19) greater than at P4 (3.4 ± 0.8%) and adult (2.6 ± 0.1%). Entry of sucrose into the CSF (Fig. 2C) in fetal pups was higher than in the brain, reaching 13.4 ± 2.2% at E16 and 9.8 ± 2.4% at E19 and was much higher compared to postnatal animals (3.2 ± 1.5% and 1.0 ± 0.6%; P4 and adult respectively), as shown in Fig. 2.

### Comparison of brain and CSF concentration ratios between the two markers

**Fig. 3:**
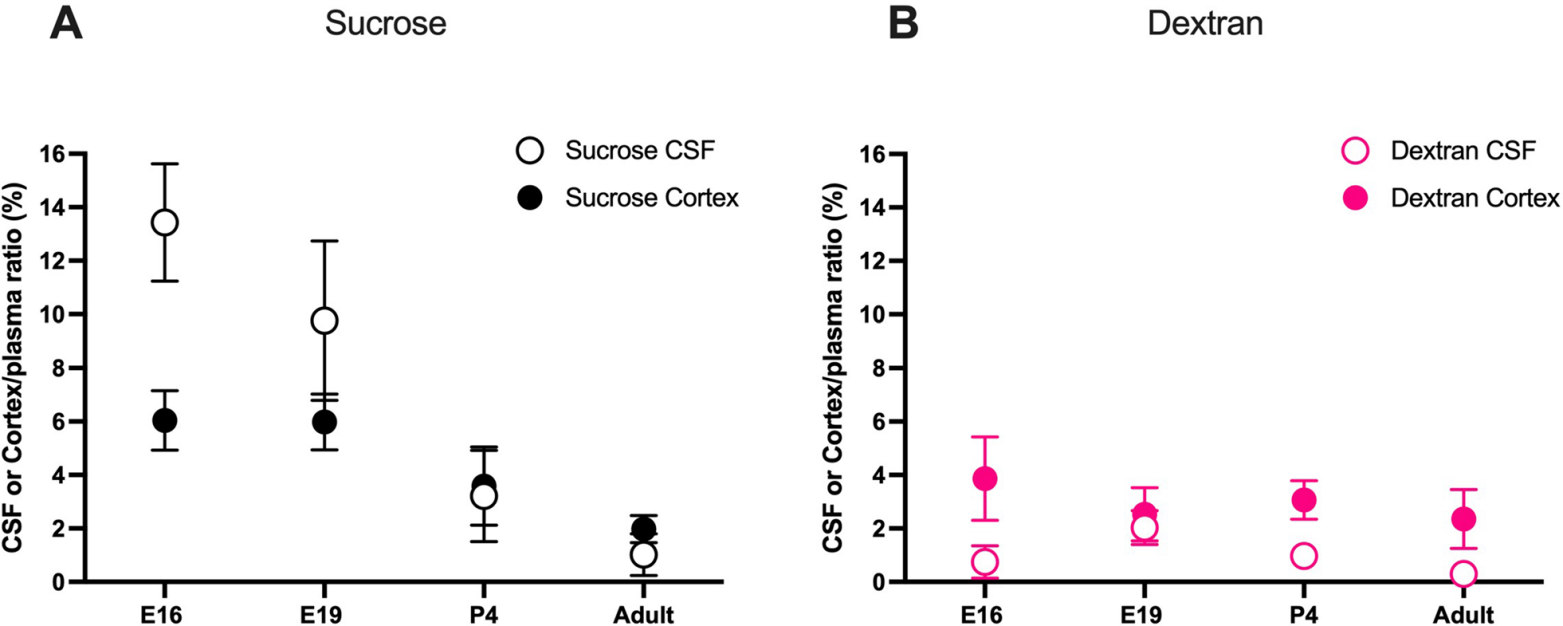
Comparison between cortex & CSF/plasma concentration ratios of the two markers. Cortex/plasma (filled●) and CSF/plasma (open○) concentration ratios (%) of sucrose (**A**) and dextran (**B**) in E16, E19, P4 and adult rats. Samples collected 5 min following i.v. injection of ^3^H-dextran or ^14^C-sucrose. Note sucrose CSF ratios were higher than brain ratios in younger animals but dextran ratios were similar at all ages. Mean ± SD, some error bars are too small to be visible; n = 3–6

Results from Figs. 1 & 2 are presented together in Fig. 3 to compare brain and CSF ratios across age groups for each marker. It is clear that sucrose ratios in the CSF were higher than in the brain in fetal animals and decreased with age (Fig. 3A). Conversely, dextran ratios in the CSF appeared to be lower than ratios in the brain in all age groups investigated. In addition, brain/plasma concentration ratios for dextran were similar across all ages. Potential explanation for these findings are considered below (see Discussion).

### Dextran and sucrose brain and CSF ratios in very short experiments

**Fig. 4:**
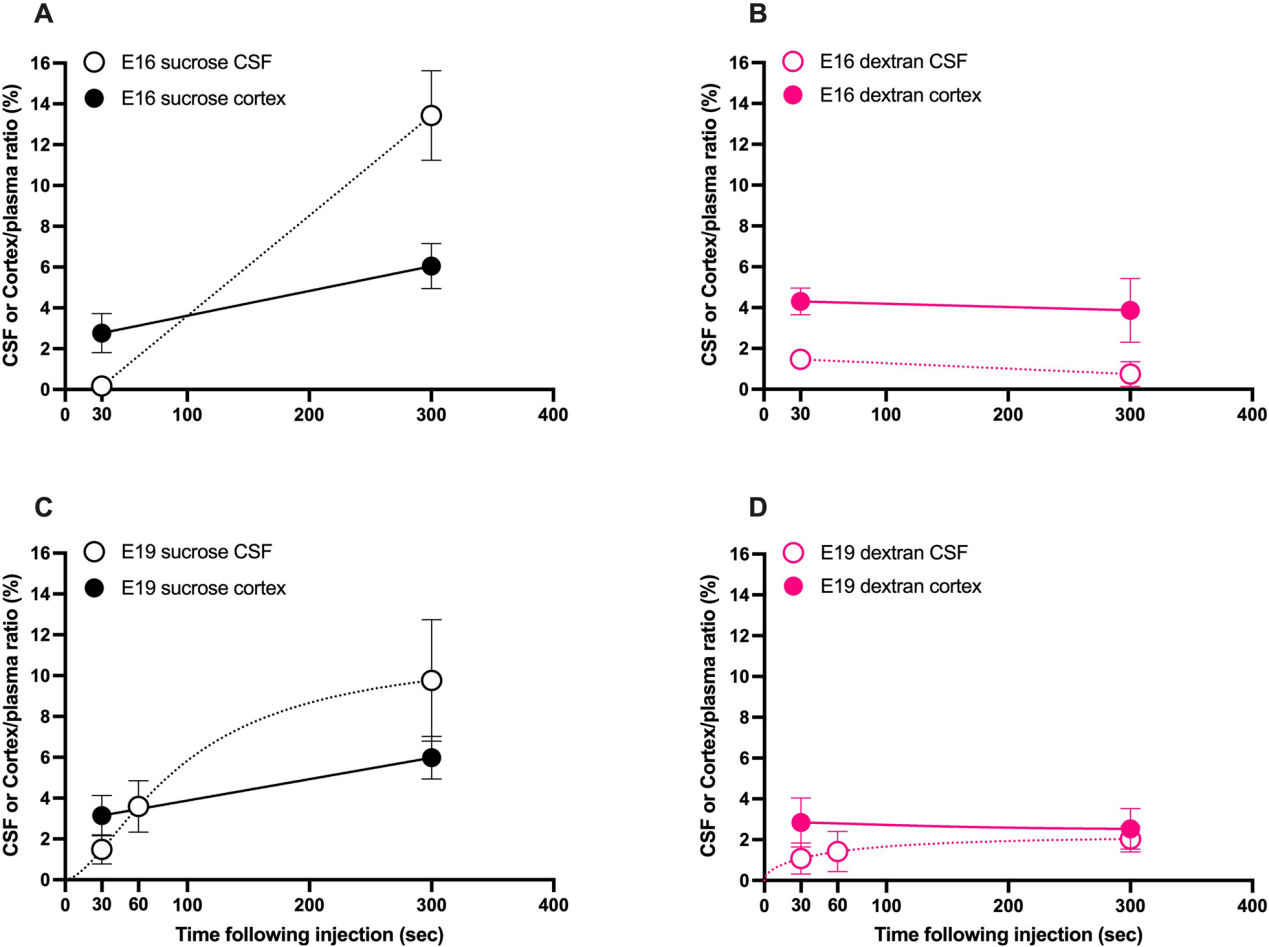
Dextran and sucrose entry into brain and CSF in shorter experiments. Cortex/plasma (filled●) and CSF/plasma (open○) concentration ratios (%) of sucrose and dextran in E16 (**A** & **B**) and E19 (**C** & **D**) pups. Samples collected 30 s, 1 min or 5 min following i.v. injection of ^3^H-dextran or ^14^C-sucrose. Note sucrose but not dextran entry in CSF increased with time in both E16 & E19. Mean ± SD where possible, n = 2–4

In a separate series of fetal experiments, injected sucrose or dextran circulation time was shortened to 30 s or 1 min before collection of samples. Sucrose ratios in both E16 & E19 brains after only 30 s were lower than those after 5 min and were in fact comparable to ratios obtained using 70 kDa dextran even after 5 min (Fig. 4A & C). Plotting results for both markers as a function of time (30 s to 5 min) showed that presence of sucrose in brain and CSF increased with time especially at E16 and to a lesser degree at E19 (Fig. 4A & C) whilst dextran ratios remained unchanged (Fig. 4B & D). In addition, at E16 30 s after sucrose injection (Fig. 4A), there were no measurable levels of the marker in the CSF and simultaneously the sucrose ratio in the brain was also lower than at 5 min and was similar to dextran ratios (Fig. 4D). This further reinforces the proposition that sucrose entry into the brain without influence from its entry via the CSF appears to reflect the true RVS, similar to dextran values obtained at all ages (Fig. 3B).

## Discussion

The present study investigated the entry into the brain and CSF of two hydrophilic markers of different molecular size, sucrose (342 Da) and dextran (70 kDa) in E16, E19, P4 and adult rats in short duration experiments (30 s to 5 min) after intravenous injection of either marker. The aim was to distinguish the two routes by which substances can enter the brain and determine the contribution of entry via the CSF (across BCSFB) to the apparent entry into the brain itself across the BBB proper. The results clearly indicate that values obtained in the brain were similar for both markers in P4 and adult rats, representing the actual cerebral RVS and confirming that sucrose is confined to the intravascular space only. On the other hand, in fetal animals, brain entry of sucrose appeared to be higher than that of dextran and higher than at postnatal ages. This indicates that some sucrose is present outside of the vasculature. It is worth noting that both markers would only distribute in the extracellular space (ECS) of the brain. In fetal brains sucrose ratios exceed dextran ratios by ~ 3.5% (E19) and ~ 2.2% (E16), so the concentration in the ECS would be 8.7% and 5.5% respectively (assuming an ECS of 40% in fetal rat brain as has been estimated for P2 [32]). In E19 pups 5 min after sucrose injection, the concentration in the ECS was similar to that in the CSF, but in E16 pups the concentration in the ECS was much lower than in the CSF (ECS <  < CSF). This finding is consistent with a number of studies reported that permeability of hydrophilic markers into brain and CSF appeared to be higher in younger animals compared to adults in several species including rat and sheep [23, 25, 33, 34]. Reported lower vascularisation in early brain development together with apparent increased permeability of the BBB has led many to a long-held erroneous belief that the BBB is immature and therefore “leaky” (Reviewed in [35]). This was in spite of the well documented morphological and physiological evidence showing that brain capillaries have functional tight junctions from the earliest stages of development [36–38]. We propose that this apparent higher entry in the immature brain of a small molecular size marker is not an indication of “immature” BBB, but instead is a result of an increased contribution of diffusion from the CSF into brain.

Apparent entry of molecules into the CSF has been demonstrated to decrease with age and appears to be inversely correlated with their size during development [34]. The possible reasons for this reduction have been discussed previously and they include increase in volume of the CSF distribution space with development and increase in rate of CSF turnover which would accelerate the removal of substances that entered the CSF [6, 39, 40]. Once the markers are in CSF, they may diffuse passively into the brain across the CSF-brain interface; however, in the developing brain the two compartments (CSF and brain) are separated by strap junctions between neuroepithelial cells [9–11]. These junctional complexes progressively disappear and are replaced by the gap junctions found in adults [41, 42]. In a study in fetal sheep, inulin (5 kDa) and sucrose (342 Da) were shown to diffuse from ventricles into the cerebral hemisphere, but transfer of larger markers such as horseradish peroxidise (40 kDa) was restricted by these strap junctions [11]. The findings of this study accord with a study using mice which demonstrated that the CSF-brain interface in pups at E19 or older allows for diffusion of biotin ethylenediamine (268 Da) from CSF into the brain, whereas movement of 70 kDa dextran is restricted until after P10 [10].

Passive diffusion of any substance is inversely related to its size, according to the Sutherland-Einstein equation [43]:

$$D=\frac{{k}_{B} T \times {10}^{13}}{{d}_{H} \pi \eta \times 3}$$

where ${k}_{B}$ is Boltzmann’s constant (1.38 J$\times {10}^{-23}$/°K), T is temperature in °K (310), ${d}_{H}$ is the molecular diameter in nm (0.94 for sucrose and 10.2 for 70 k dextran [44]), $\eta$ is viscosity in Pa⋅s which is assumed to be similar to that of water at the same temperature (0.00069)[45]. The free diffusion coefficients (D, cm^2^/s) are calculated to be 0.7 $\times {10}^{-5}$ & 0.0064 $\times {10}^{-5}$ for sucrose and 70 k dextran respectively. Using these diffusion coefficients and Fick’s second law [43]:

$${r}^{2}=2 \times D t$$

where r is distance from origin after a period of time, t, it can be estimated that in 5 min sucrose would passively diffuse approximately 648 μm while dextran would only diffuse 62 μm (see Table 3 below). Although this estimation applies only to diffusion distance in free medium, which is different to how molecules would move in biological environments such as the brain interstitial fluid, the emphasis is on the relative comparison of the potential diffusional distance between sucrose and dextran [32]. This suggests that there would be a substantial transfer of sucrose from CSF to brain without restriction between the two compartments, especially in smaller animals where the volume and dimensions of the brain are relatively less than in older animals. Approximately, the diffusion distance of sucrose in 5 min is about 88% of the thickness of the cortex in an E19 animal, compared to 30% in adult brains (Table 3). Moreover, the concentration of sucrose in CSF was greater during fetal stages than in adults (9.8 ± 2.4% vs 1.0 ± 0.6%; Fig. 2C), so higher cortex & brainstem ratios in younger animals could probably be explained by entry from CSF. On the other hand, diffusion of 70 kDa dextran from CSF into the brain can be expected to be restricted by the CSF-brain barrier until late postnatal period. In adults, its low CSF accumulation and slow diffusion would probably have little impact on the adult brain/plasma ratios. Further in support of this explanation are results from the shorter experiments in fetal animals (Fig. 4); where in 30 s sucrose had barely entered CSF whilst brain ratios at that time had reached levels similar to the dextran brain ratios. Between 30 s and 5 min, more sucrose would have entered the brain via CSF as indicated by the greater increase in CSF/plasma ratio compared to brain.

**Table 3:** Calculated approximate diffusion distance of dextran and sucrose in short experiments

	30 s (μm)	5 min (μm)	5 min diffusion distance/E19 cortex thickness (%)	5 min diffusion distance/Adult cortex thickness (%)
Sucrose	205	648	88%	30%
Dextran	20	62	×	3%

Distances calculated as described in text. Thickness of cortex in E19 and adult rats provided in Additional file 1: Table S3. × indicates that the route into the brain is restricted against 70 kDa dextran in E19 due to strap junctions [10]

Any movement of hydrophilic substances into and out of the brain could also be affected by the glymphatic system. Information on the role of the glymphatic system is limited especially in the developing brain. One study in mice clearly showed that the glymphatic system is not functional in fetal and neonatal brain [46]. In addition, the turnover of CSF in the fetal and neonatal brain is very low [40]. Thus, in early stages of brain development the glymphatic system is not likely to influence the entry and clearance of sucrose in the CSF and brain. Even when the glymphatic system does become functional in the postnatal period [46], the very short times used in the present study suggest that its effects are likely to be very limited.

Distances calculated as described in text. Thickness of cortex in E19 and adult rats provided in Additional file 1: Table S3. X indicates that the route into the brain is restricted against 70 kDa dextran in E19 due to strap junctions [10].

Estimation of RVS in the brain is important in in vivo permeability experiments such as studies examining the extent of entry of drugs into the brain, especially those with low BBB permeability such as hydrophilic molecules. The RVS should be estimated in order to more accurately evaluate the amount of the drug or other molecules that has actually permeated out of vessels [25–27, 29–31]. In order to determine the residual vascular space in vivo, markers of very low permeability into brain are generally used [25, 26]. Inert and hydrophilic markers are chosen because they are not typically subject to metabolism, active transport or binding with native, circulating plasma proteins in a short experimental period [26, 43, 47, 48]. To minimise the effects of markers leaving blood vessels, resulting in an overestimated RVS, tissues are either collected shortly after introduction of markers or a time course of ratios is constructed in order to extrapolate to an earlier time point before there has been significant loss into the tissues [34, 47–49]. In the present study it has been shown that 70 kDa dextran, compared to sucrose (342 Da), appears to be a more suitable marker for estimation of brain RVS as it is less affected by diffusion from the CSF. Very short experimental time points were also used to further ensure that dextran is confined within blood vessels and the molecule was not likely to be subjected to metabolism. Furthermore, all animals were injected via the same route, intravenously, to allow for rapid distribution through circulation and better comparison between age groups.

One limitation in the current experiment design is that concentrations in brain were related to plasma levels, resulting in the reflection of plasma space in the brain rather than actual blood volume. It has been examined in detail previously that the blood haematocrit in rat brain can be different to the haematocrit in peripheral blood, and it can vary in different regions of the brain [50, 51]. Estimations of the two markers used in the present study did not take into account the volume of red blood cells and therefore the RVS might be slightly overestimated, but information on haematocrit in the developing rat brain has not been reported. The RVS appears to be similar in all age groups investigated contrary to the findings that extensive vascularisation, including increase in endothelial cell number, capillary length and surface area, occur afterbirth in the rat as described earlier. Due to very small size of fetuses, CSF was collected in a different location for E16 pups (lateral ventricle instead of *cisterna magna*). However, since the overall internal volume of the CSF system is small, samples of CSF obtained in our experiments would have drained most of the volume of the internal CSF system and are therefore representative of a mixture of lateral ventricle, 3^rd^ and 4th ventricular CSF [6, 39].

The other limitation is the number of fetal animals used in this study. However, the key finding is a distinct developmental decrease in entry of sucrose between the fetal ages (E16 & E19) and postnatal animals (P4 and adults) as shown in Fig. 2. Within the fetal group, the higher ratios were similar between E16 and E19 and within the postnatal group, the lower ratios were similar from P4 to adult. Differences between any fetal and postnatal ages are well powered (80% at an alpha of 0.01). For Fig. 1 dextran space in all age groups are highly similar which is expected from previous studies in literature (discussed above). In the short time course experiments (Fig. 4), the number of fetal animals at each age is low, but all ages contribute to the trend lines/curves. The emphasis of these graphs is to illustrate the direction of change in ratios over time rather than a statistical comparison between individual timepoints.

## Conclusions

In conclusion, due to higher entry into the CSF across the BCSFB and subsequent diffusion from CSF into the brain, a small hydrophilic marker such as sucrose may appear to have a higher brain space during development compared to a larger marker 70 kDa dextran. Very short experiments demonstrated that without contribution from the CSF, sucrose brain/plasma ratios in the fetus were lower and were similar to those obtained using 70 kDa dextran. In addition, the results demonstrated that in the rat the RVS in the brain measured with 70 kDa ^3^H-dextran did not change from E16 to adult.

## Supplementary Information

**Additional file 1: Table S1**. Values for dextran experiments. **Table S2.** Values for sucrose experiments. **Table S3**. Vertical Thickness of cortex in E19 & Adult brain estimated histologically.

## Data Availability

All data generated or analysed during this study are included in this published article and its supplementary information files.

## Abbreviations

BBB: Blood–brain barrier
BCSFB: Blood-cerebrospinal fluid barrier
CNS: Central nervous system
CSF: Cerebrospinal fluid
Da: Dalton
DPM: Disintegration per minute
E: Embryonic Day
iv: Intravenous
min: Minute
P: Postnatal day
SD: Standard deviation
sec: Second
RVS: Residual vascular space
