# Supplementary material for: Age dependent contribution of entry via the CSF to the overall brain entry of small and large hydrophilic markers
Source: Fluids Barriers CNS. 2022 Nov 14;19:90. doi: 10.1186/s12987-022-00387-z (PMC9661750; doi:10.1186/s12987-022-00387-z)
Supplement: Supplementary file 1 — Additional file 1: Table S1. Values for dextran experiments. Table S2. Values for sucrose experiments. Table S3. Vertical Thickness of cortex in E19 & Adult brain estimated histologically. [file 12987_2022_387_MOESM1_ESM.docx]

|  | Cortex (n) | Brainstem (n) | CSF (n) | Total n |
| --- | --- | --- | --- | --- |
| E16 – 30 sec | 4.3 ± 0.7 (3) | - | 1.2, 1.7 (2) | 5 |
| E16 – 5 min | 3.8 ± 1.6 (4) | 3.2 ± 1.2 (4) | 0.7 ± 0.6 (3) | 7 |
| E19 – 30 sec | 2.8 ± 1.2 (3) | - | 1.1 ± 0.8 (4) | 7 |
| E19 – 1 min | - | - | 1.4 ± 1.0 (4) | 4 |
| E19 – 5 min | 2.5 ± 1.0 (6) | 4.2 ± 1.3 (6) | 2.0 ± 0.6 (4) | 10 |
| P4 – 5 min | 3.1 ± 0.6 | 3.6 ± 0.3 | 1.0 ± 0.1 | 4 |
| Adult– 5 min | 2.4 ± 1.0 | 4.2 ± 0.5 | 0.3 ± 0.2 | 4 |

**Additional file Table 1.** **Values for dextran experiments**

Cortex/plasma, Brainstem/plasma and CSF/plasma concentration ratios (%) of ^3^H-dextran in E16, E19, P4 and adult animals. Samples collected 30 seconds, 1 minute or 5 minutes following i.v. injection. For fetal experiments, brain regions and CSF were collected from separate individuals due to small size, in all postnatal rats brain and CSF samples were collected from the same animal; (n) indicates numbers of samples. Mean ± SD for n>2. Total n is total number of animals.

**Additional file Table 2. Values for sucrose experiments**

|  | Cortex (n) | Brainstem (n) | CSF (n) | Total n |
| --- | --- | --- | --- | --- |
| E16 – 30 sec | 2.8 ± 1.0 (3) | - | 0.2 ± 0.3 (3) | 6 |
| E16 – 5 min | 6.0 ± 1.1 (3) | 6.5 ± 1.3 (3) | 13.4 ± 2.2 (3) | 6 |
| E19 – 30 sec | 3.2 ± 1.0 (3) | - | 1.1 ± 0.8 (4) | 7 |
| E19 – 1 min | - | - | 3.1 ± 1.5 (4) | 4 |
| E19 – 5 min | 6.0 ± 0.9 (5) | 7.0 ± 1.4 (5) | 9.8 ± 2.4 (3) | 8 |
| P4 – 5 min | 3.6 ± 1.3 | 3.4 ± 0.8 | 3.2 ± 1.5 | 6 |
| Adult– 5 min | 2.0 ± 0.4 | 2.6 ± 0.1 | 1.0 ± 0.6 | 3 |

Cortex/plasma, Brainstem/plasma and CSF/plasma concentration ratios (%) of ^14^C-sucrose in E16, E19, P4 and adult animals. Samples collected 30 seconds, 1 minute or 5 minutes following i.v. injection. For fetal experiments, brain regions and CSF were collected from separate individuals due to small size, in all postnatal rats brain and CSF samples were collected from the same animal; (n) indicates numbers of samples. Mean ± SD. Total n is total number of animals.

**Additional file Table 3. Vertical Thickness of cortex in E19 & Adult brain estimated histologically.**

|  | Mean length (μm) | n |
| --- | --- | --- |
| E19 brain | 739.4 | 5 |
| Adult brain | 2138.8 | 4 |

From previous studies whole brains from PFA-perfused adult rats and E19 fetuses were extracted and immersed in Bouin’s fixative for 24 hours followed by dehydration in ethanol. They were then embedded in paraffin wax and 5 μm slices were coronally sectioned using a microtome. Slides were dewaxed and stained with haematoxylin and eosin. Photographs of sections were taken using an Olympus bright field microscope and distance between lateral ventricle and dorsal brain surface was measured using ImageJ.
